# Jasmonic Acid and Ethylene Signaling Pathways Regulate Glucosinolate Levels in Plants During Rhizobacteria-Induced Systemic Resistance Against a Leaf-Chewing Herbivore

**DOI:** 10.1007/s10886-016-0787-7

**Published:** 2016-11-15

**Authors:** Nurmi Pangesti, Michael Reichelt, Judith E. van de Mortel, Eleni Kapsomenou, Jonathan Gershenzon, Joop J. A. van Loon, Marcel Dicke, Ana Pineda

**Affiliations:** 1 Laboratory of Entomology, Wageningen University, P.O. Box 16, 6700 AA Wageningen, The Netherlands; 2 Department of Biochemistry, Max Planck Institute for Chemical Ecology, 07745 Jena, Germany; 3 Laboratory of Phytopathology, Wageningen University, P.O. Box 16, 6700 AA Wageningen, The Netherlands; 4 HAS University of Applied Sciences, 5911 KJ Venlo, The Netherlands; 5 Department of Terrestrial Ecology, Netherlands Institute of Ecology (NIOO-KNAW), PO Box 50, 6700 AB Wageningen, The Netherlands

**Keywords:** Camalexin, Glucosinolates, Induced systemic resistance, Plant growth-promoting rhizobacteria, Signaling pathways

## Abstract

Beneficial soil microbes can promote plant growth and induce systemic resistance (ISR) in aboveground tissues against pathogens and herbivorous insects. Despite the increasing interest in microbial-ISR against herbivores, the underlying molecular and chemical mechanisms of this phenomenon remain elusive. Using *Arabidopsis thaliana* and the rhizobacterium *Pseudomonas simiae* WCS417r (formerly known as *P. fluorescens* WCS417r), we here evaluate the role of the JA-regulated MYC2-branch and the JA/ET-regulated ORA59-branch in modulating rhizobacteria-ISR to *Mamestra brassicae* by combining gene transcriptional, phytochemical, and herbivore performance assays. Our data show a consistent negative effect of rhizobacteria-mediated ISR on the performance of *M. brassicae.* Functional JA- and ET-signaling pathways are required for this effect, as shown by investigating the knock-out mutants *dde2-2* and *ein2-1*. Additionally, whereas herbivory mainly induces the MYC2-branch, rhizobacterial colonization alone or in combination with herbivore infestation induces the ORA59-branch of the JA signaling pathway. Rhizobacterial colonization enhances the synthesis of camalexin and aliphatic glucosinolates (GLS) compared to the control, while it suppresses the herbivore-induced levels of indole GLS. These changes are associated with modulation of the JA-/ET-signaling pathways. Our data show that the colonization of plant roots by rhizobacteria modulates plant-insect interactions by prioritizing the JA/ET-regulated ORA59-branch over the JA-regulated MYC2-branch. This study elucidates how microbial plant symbionts can modulate the plant immune system to mount an effective defense response against herbivorous plant attackers.

**Electronic supplementary material:**

The online version of this article (doi:10.1007/s10886-016-0787-7) contains supplementary material, which is available to authorized users.

## Introduction

Plants as primary producers in terrestrial ecosystems are exposed to various attackers, with insect herbivores among the most important ones. To survive, plants have evolved physical and chemical barriers as defenses against insect herbivores. Upon recognition of insect elicitors, plants use hormones that regulate signaling pathways to reprogram their transcriptome and metabolome, thus strengthening their defense (Bodenhausen and Reymond 2007; De Vos et al. 2005; Reymond et al. 2004). In Brassicaceous plants, including *Arabidopsis thaliana*, glucosinolates (GLS) are the main defensive compounds that confer plant resistance against insect herbivores (Beekwilder et al. 2008; Howe and Jander 2008; Mewis et al. 2006; Müller et al. 2010). The two most abundant classes of GLS are aliphatic and indolic GLS, depending on whether the side chain is derived from the amino acid methionine or tryptophan, respectively (Halkier and Gershenzon 2006). Feeding by specialist and generalist leaf-chewing insects triggers enhanced synthesis of aliphatic and indolic GLS (Kos et al. 2012; Verhage et al. 2011). More recent studies show that other compounds, such as camalexin, a brassicaceous indolic phytoalexin, also contribute to plant resistance against herbivores (Kettles et al. 2013; Kusnierczyk et al. 2008; Prince et al. 2014; Schlaeppi et al. 2008). Unraveling how plant signaling pathways and crosstalk between pathways regulate the synthesis of defensive compounds in the context of multitrophic interactions has only just begun.

The signaling pathway regulated by the plant hormone jasmonic acid (JA) is the core pathway regulating resistance to leaf-chewing herbivores (Erb et al. 2012; Farmer and Ryan 1992; Howe and Jander 2008; Kessler and Baldwin 2002), through formation of physical barriers such as trichomes and enhanced synthesis of defensive compounds such as GLS (Erb et al. 2012; Howe and Jander 2008). The JA-signaling pathway has two branches that cross-communicate with other hormonal pathways, such as the ethylene (ET) and abscisic acid (ABA) pathways through the use of common transcription factors (Kazan and Manners 2008; Lorenzo and Solano 2005). The transcription factor ORA59 is one of the main integrators of the JA- and ET-signaling pathways (Lorenzo et al. 2003; Pre et al. 2008), whereas transcription factor MYC2 is one of the main integrators of JA- and ABA-signaling pathways (Abe et al. 2002; Vos et al. 2013). Each of these two transcription factors activates different sets of JA-responsive genes (Kazan and Manners 2013; Lorenzo and Solano 2005; Pieterse et al. 2012). In *A. thaliana*, MYC2 regulates the biosynthesis of defensive compounds such as camalexin and GLS (Kazan and Manners 2013; Schweizer et al. 2013). In line with this, feeding by the leaf-chewing insects *Pieris rapae* and *Helicoverpa armigera* induces the MYC2-branch and enhances the expression of the JA-responsive gene *Vegetative Storage Protein 2* (*VSP2*) (Verhage et al. 2011). The MYC2-branch also has an important function in mediating the ability of beneficial microbes to prime plant defenses to pathogens (Pozo et al. 2008). An intriguing question then is whether the MYC2-branch also plays a role in the ability of beneficial microbes to increase plant resistance to insect herbivores.

Plants host a diversity of microbes, including beneficial microbes in the rhizosphere that can affect plant defense and growth. The influence of beneficial microbes on plant defense and growth is mediated by their effect on plant signaling pathways that not only regulate microbial colonization but also trigger significant changes in plant gene expression, metabolism, and physiology (Cartieaux et al. 2008; Van de Mortel et al. 2012; Verhagen et al. 2004; Weston et al. 2012). Interestingly, several species of root-associated microbes from the genera *Pseudomonas*, *Bacillus*, and *Trichoderma* enhance plant immunity, through a mechanism called induced systemic resistance (ISR), known to inhibit growth and development of various insect herbivores and pathogens (Pangesti et al. 2015; Pineda et al. 2010; Song et al. 2013; Valenzuela-Soto et al. 2010). Intact JA and ET hormonal signaling pathways are required to induce ISR by several root-associated microbes such as *P. fluorescens* WCS417r against pathogens (Pieterse et al. 1998). Based on the whole genome sequence comparison, this rhizobacterium isolate recently has been renamed into *Pseudomonas simiae* WCS417r (Berendsen et al. 2015). However, it is unknown if intact JA and ET signaling pathways also regulate ISR against insect herbivores. Furthermore, it remains to be elucidated how plants regulate chemical defense against insect herbivores upon colonization by root-associated beneficial microbes.

The present study investigates how colonization by the rhizobacterium *P. simiae* WCS417r affects plant defense against the leaf-chewing insect *Mamestra brassicae*. Previous studies have found that this rhizobacterium triggers an enhanced expression of the JA-regulated gene *LOX2* and the JA/ET-regulated genes *PDF1.2* and *HEL* upon feeding by the generalist caterpillars *M. brassicae* and *Spodoptera exigua* (Pangesti et al. 2015; Van Oosten et al. 2008). However, whether the JA-regulated MYC2-branch or the JA/ET-regulated ORA59-branch is modulating plant defense in rhizobacteria-mediated ISR against insects is unknown. To investigate this, gene transcription, plant chemistry, and performance of the herbivore *M. brassicae* were analyzed *in vitro* in wild type *A. thaliana* Col-0 and in mutants defective in the JA pathway, *i.e.*, *dde2-2* and *myc2*, in the ET pathway, *i.e.*, *ein2-1*, and in the JA/ET pathway, *i.e.*, *ora59.* We hypothesized that rhizobacteria-treatment of plants 1) triggers enhanced expression of the JA/ET-regulated genes *ORA59* and *PDF1.2* and of the JA-regulated genes *MYC2* and *VSP2* upon feeding by *M. brassicae,* 2) increases the synthesis of glucosinolates and camalexin upon feeding by *M. brassicae*, and 3) results in stronger resistance to *M. brassicae via* the JA- and ET-signaling pathways.

## Methods and Materials

### Rhizobacterium *Pseudomonas simiae* WCS417r, Growing Conditions, and Quantification

The rifampicin-resistant, non-pathogenic epiphyte rhizobacterium strain *P. simiae* WCS417r (abbreviated as *Ps.* WCS417r) was used. Rhizobacteria were grown on King’s B (KB) medium agar plates containing rifampicin (25 μg ml^−1^) for 48 h at 28°C (Pieterse et al. 1996). Prior to inoculation on plant roots, a single colony of the strain was transferred to KB liquid medium amended with rifampicin as indicated above, and was grown in an incubator shaker for 24 h at 200 rotations per minute (rpm) at 25°C. The bacterial cells were collected, re-suspended in 10 mM MgSO_4_, and washed three times with 10 mM MgSO_4._ Afterwards, the bacterial cells were re-suspended in 10 mM MgSO_4_ and adjusted to a cell density of 1x10^9^ colony forming units (cfu) ml^−1^ (OD660 = 1.0).

Colonization of *A. thaliana* roots by *Ps.* WCS417r was quantified in wild type plants and mutants to confirm that the colonization met the required threshold for ISR of 10^5^ cfu.g^−1^ root (Raaijmakers et al. 1995). The rhizobacteria quantification was done following the method described in Pangesti et al. (2015), with slight modification. Roots were harvested, weighed, and shaken vigorously for 1 min in 10 ml of 10 mM MgSO_4_ containing 0.5 g of glass beads (425–600 μm, Sigma-Aldrich). Proper dilutions were plated onto KB agar medium supplemented with 25 μg ml^−1^ rifampicin to select for rifampicin-resistant fluorescent *Pseudomonas* spp. (Pieterse et al. 1998). The dilution plates were incubated for 48 h at 28°C, after which the number of cfu per mg root fresh weight was determined.

### *Mamestra brassicae* Rearing

The generalist insect herbivore *M. brassicae* L. (Lepidoptera: Noctuidae; Cabbage moth) was reared on *Brassica oleracea* L. var. *gemmifera* cv. Cyrus (Brussels sprouts) in a climate chamber (22 ± 2°C, 40 - 50% RH, 16:8 h photo:scotophase). Newly-emerged larvae were used in the experiments.

### Cultivation of *A. thaliana* Col-0 *in vitro*

*Arabidopsis thaliana* Col-0 plants were surface-sterilized and grown *in vitro* following a method described in Van de Mortel et al. (2012). In this study, *A. thaliana* Col-0 and mutants defective in the JA signaling pathway (*dde2-2*, *myc2*) and in the JA/ET signaling pathway (*ein2-1*, *ora59*) were used. Mutant *dde2-2* is defective in ALLENE OXIDE SYNTHASE, a key enzyme in the JA-biosynthesis pathway (Von Malek et al. 2002), mutant *myc2* is defective in transcription factor MYC2/JIN1 and is activated by the JA-signaling pathway (Hiruma et al. 2011). Mutant *ein2-1* is defective in the nuclear protein ETHYLENE INSENSITIVE 2–1, a central component of the ET-signaling pathway (Guzman and Ecker 1990), mutant *ora59* is defective in transcription factor ORA59 that is involved in the JA/ET-signaling pathways (Pre et al. 2008). A total of 12 seeds of the same line were sown on square plates (100 × 100 × 20 mm) (SARSTEDT, Nümbrecht, Germany) containing 50 ml of half-strength Murashige and Skoog (MS) medium (Murashige and Skoog 1962), and seeds were incubated for 7 d in a growth chamber at 21 ± 2°C, 60% relative humidity (RH), 16:8 h L:D cycle, and 90 ± 1 μmol m^−2^ s^−1^ light intensity (SYLVANIA, GRO-LUX®, Germany). Seven-d-old plant seedlings were root tip-inoculated with 2 μl of *Ps*. WCS417r cell suspension (10^9^ CFU ml^−1^). For control treatment, plant seedlings were mock-inoculated with 2 μl of MgSO_4_ solution. After root inoculation, plants were incubated for an additional 7 d in the same conditions as described above. Fourteen-d-old plants were used in the experiments.

### Experiment 1. Expression of Marker Genes of the ORA59- and MYC2-Branch During Rhizobacteria-Mediated ISR

We evaluated gene expression of JA/ET-regulated genes *ORA59* and *PDF1.2* and of the JA-regulated genes *MYC2* and *VSP2* in *A. thaliana* Col-0 and mutants *myc2* and *ora59*. The four treatments, consisting of control plants (C), rhizobacteria-treated plants (R), control plants infested with *M. brassicae* (CM), rhizobacteria-treated plants infested with *M. brassicae* (RM) were arranged for each line. In herbivory treatments, two larvae per plant were placed on the leaves. For each treatment, four to five biological replicates were used, each consisting of pooled leaves taken from four to five plates (each containing 11 to 12 seedlings) to ensure sufficient plant material for gene transcript analysis. Leaves were harvested at 24 h after insect infestation (hpi). Leaves of uninfested plants were treated and harvested similarly as those of infested plants. Leaf samples were frozen immediately in liquid nitrogen and stored at −80°C for further RNA extraction. Using the same batch of plants, performance of the caterpillars feeding on *Arabidopsis* wild type Col-0, and on the *myc2* and *ora59* mutants, and plant biomass were assessed as described below.

Leaf samples were ground in liquid nitrogen, and total RNA was extracted and purified following the protocol of RNeasy plant mini kit (Qiagen, Hilden, Germany). Measurement of RNA quality and procedure of cDNA synthesis followed methods described in Pangesti et al. (2015). Sequences of the primers used in this study for the genes *MYC2*/*JASMONATE INSENSITIVE1* (*MYC2*), *VEGETATIVE STORAGE PROTEIN 2* (*VSP2*), *OCTADECANOID-RESPONSIVE ARABIDOPSIS 5*9 (*ORA59*), *PLANT DEFENSIN 1.2* (*PDF1.2*) are provided in the Supplementary materials. Efficiency of each primer was determined before qRT-PCR analysis (CFX96™ Real-Time System, BIO-RAD, Hercules, CA, USA). Thermal cycling conditions consisted of 95°C for 3 min, followed by 40 cycles of 95°C for 15 s and 62°C for 45 s. For each primer pair, controls without addition of template were included to confirm that primer dimers were not interfering with detection of amplification. The transcript level for each tested gene was calculated relative to the reference genes *ELONGATION FACTOR 1α* (*EF1α*) and *F-BOX FAMILY PROTEIN* (*FBOX*) using the 2^ΔΔCt^ method (Livak and Schmittgen 2001).

### Performance of *M. brassicae* and Measurement of Plant Biomass

From the same batch of plants as for gene transcript analysis, additional plates were arranged to evaluate herbivore performance and plant biomass. Larvae that were feeding on *Arabidopsis* wild type Col-0, *myc2*, or *ora59* were weighed at 4 d post infestation (dpi), to the nearest 0.001 mg on a microbalance (CP2P, Sartorius AG, Germany). Afterwards, a pool of plant leaf material left in each replicate (squared plates) from control plants infested with *M. brassicae* (CM), rhizobacteria-treated plants infested with *M. brassicae*, as well as control plants (C) and rhizobacteria-treated plants (R) were weighed to the nearest 0.1 mg (Mettler Toledo, Switzerland). Bioassays were performed in a growth chamber under similar conditions as described for plant cultivation.

### Experiment 2. Changes in Glucosinolate and Camalexin Levels During Rhizobacteria-ISR in the JA and ET-Defective Mutants dde2-2 and ein2-1

We evaluated the concentrations of glucosinolates (GLS) and camalexin in *A. thaliana* Col-0, in the JA-biosynthesis defective mutant *dde2-2* and in the ET-signaling defective mutant *ein2-1*. Using the same batch of plants, performance of the caterpillars feeding on *Arabidopsis* wild type Col-0, *dde2-2*, *ein2-1*, and plant biomass were measured as described above.

### Glucosinolate and Camalexin Analysis

For glucosinolate (GLS) and camalexin analysis, four to five biological replicates were used, each consisting of pooled leaves taken from four to five plates (each containing 11 to 12 seedlings) to ensure sufficient material was collected for chemical analysis. Leaves were harvested at 4 dpi. Leaves of uninfested plants were treated and harvested at similar time points as those of infested plants. Leaf samples were frozen immediately in liquid nitrogen and stored at −80°C for further analysis. Leaf samples were ground to a fine powder in liquid nitrogen and then lyophilized for 48 h at −80°C and pressure of < 10 mB.

Approximately 20 mg of lyophilized tissue were weighed for glucosinolate (GLS) analysis, and the exact weight of the tissue was recorded and used to calculate the GLS concentration. The GLS were extracted with 1 ml of 80% methanol solution containing 0.05 mM intact 4-hydroxybenzylglucosinolate as internal standard, and analyzed by HPLC-UV as described in Burow et al. (2006). Camalexin was analyzed in the flow-through samples resulting from the extraction procedure for GLS analysis. In GLS extraction, the raw extract was loaded onto DEAE Sephadex, the resulting flow-through when loading the extract was collected in a 96 deep-well plate and directly analyzed by LC-MS/MS.

### Experiment 3. Changes in Glucosinolates and Camalexin During Rhizobacteria-Mediated ISR in the Transcription Factor Defective Mutants myc2 and ora59

We evaluated concentrations of GLS and camalexin in *A. thaliana* Col-0, in the ORA59-branch mutant *ora59* and in the JA-regulated MYC2-branch mutant *myc2* plants as described for experiment 2. Using the same batch of plants, performance of the caterpillar feeding on *Arabidopsis* wild type Col-0, *ora59*, *myc2*, and plant biomass of all lines were measured as described above. Analysis of GLS and camalexin content in plant shoots was performed as described for experiment 2.

### Statistical Analysis

Gene expression data were transformed [log(x + 1)] and analyzed with one-way ANOVA to compare treatments within each line, whereas two-way ANOVA was used to compare treatments between lines. Glucosinolate data were analyzed with multivariate Projection to Latent Structures-Discriminant Analysis (PLS-DA) (SIMCA P + 12.0, Umetrics AB, Umeå, Sweden). Analysis of individual and total aliphatic and indolic GLS were analyzed with one-way ANOVA to compare treatments within each line, whereas two-way ANOVA was used to compare treatments between lines. Camalexin data were log-transformed and analyzed with one-way ANOVA to compare treatments within each line, whereas two-way ANOVA was used to compare treatments between lines. *Mamestra brassicae* performance data were analyzed with Linear Mixed Models (LMMs) within each line, with treatment as fixed factor and plate as random factor. Effect of rhizobacterial colonization on *M. brassicae* performance also was assessed between lines. Data of plant shoot and root biomass were analyzed with one-way ANOVA to compare treatments within each line, whereas two-way ANOVA was used to compare treatments between lines. Results of two-way analysis are reported in Table 1.

**Table 1:** Effect of the factors treatment, line and their interaction term on levels of transcripts and defensive chemicals, as well as on herbivore performance

	Treatment			Line			Treatment x Line		
	*df*	*F*	*P*	*df*	*F*	*P*	*df*	*F*	*P*
Gene transcription: Col-0, *myc2*, *ora59* ^a^									
*MYC2*	3, 50	5.75	0.002	2, 50	28.50	< 0.001	6, 50	3.09	0.014
*VSP2*	3, 50	100.62	< 0.001	2, 50	1.85	0.170	6, 50	0.67	0.677
*ORA59*	3, 50	23.31	< 0.001	2, 50	27.25	< 0.001	6, 50	3.37	0.009
*PDF1.2*	3, 50	115.18	< 0.001	2, 50	65.32	< 0.001	6, 50	65.32	< 0.001
Chemistry^a^ and herbivore performance^b^: Col-0, *dde2-2*, *ein2-1*									
Aliphatic GLS	3, 59	18.59	< 0.001	2, 59	98.94	< 0.001	6, 59	8.83	< 0.001
Indole GLS	3, 59	166.68	< 0.001	2, 59	224.12	< 0.001	6, 59	45.18	< 0.001
Camalexin	3, 59	58.19	< 0.001	2, 59	17.65	< 0.001	6, 59	3.34	0.008
Herbivore	1, 104	7.06	0.009	2, 104	20.16	< 0.001	2, 104	0.74	0.692
Chemistry^a^ and herbivore performance^b^: Col-0, *myc2*, *ora59*									
Aliphatic GLS	3, 59	18.99	< 0.001	2, 59	5.63	0.001	6, 59	0.32	0.92
Indole GLS	3, 59	60.13	< 0.001	2, 59	6.58	0.003	6, 59	2.81	0.02
Camalexin	3, 59	37.88	<0.001	2, 59	12.89	< 0.001	6, 59	0.80	0.575
Herbivore	1, 484	45.93	< 0.001	1, 484	7.12	0.029	1, 484	0.22	0.897

^a^Two-way ANOVA; ^b^Linear Mixed Model, *F*-value represents Wald statistic

## Results

### Rhizobacterial Colonization of *Arabidopsis thaliana* Modifies Plant Signaling by Prioritizing Expression of Genes in the ORA59-Branch Over Those in the MYC2-Branch

**Fig 1:**
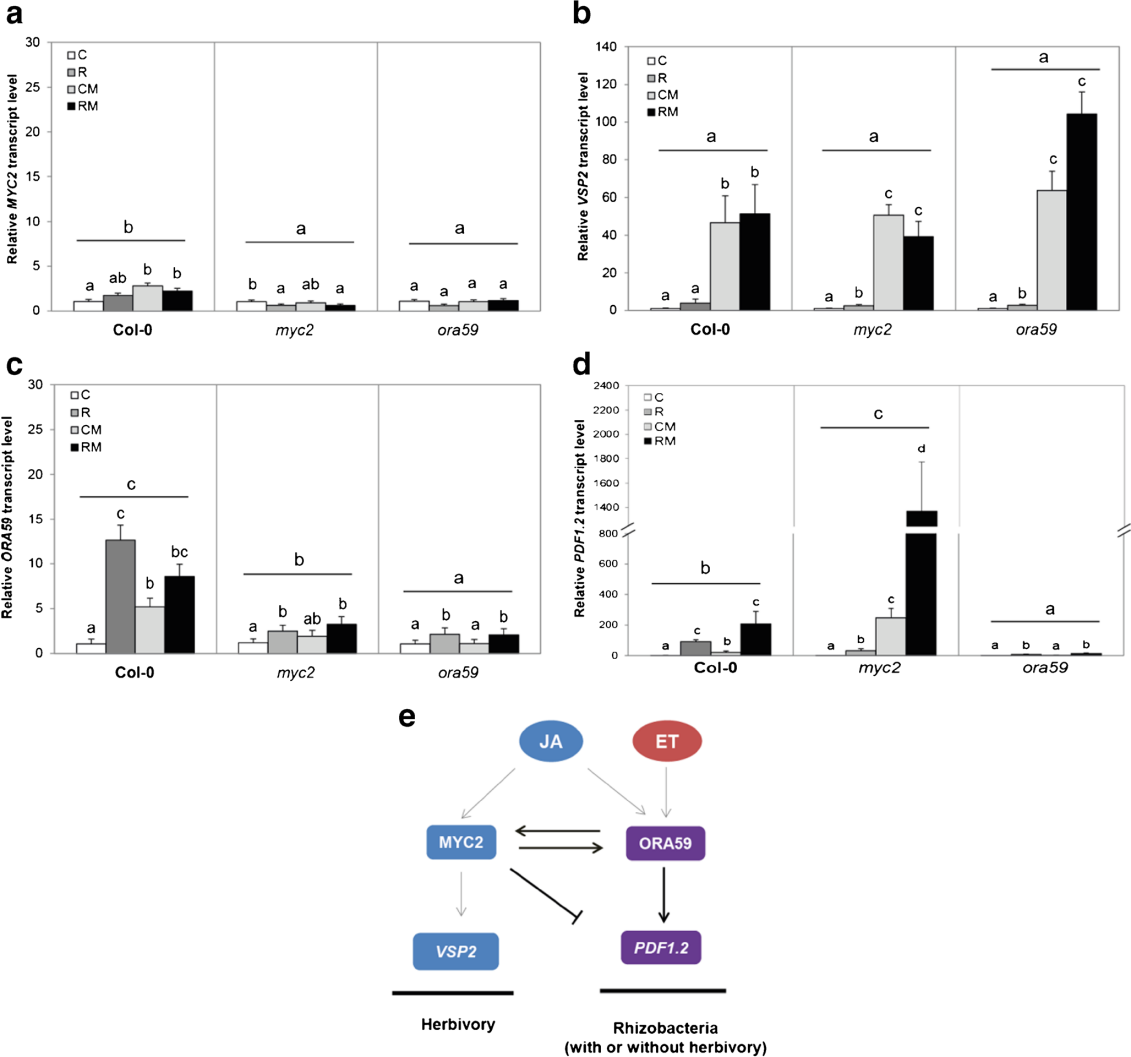
Relative transcript levels of *MYC2* **a**, *VSP2* **b**, *ORA59* **c**, and *PDF1.2* **d**. Expression of *MYC2* and *VSP2* genes are activated by the jasmonic acid (JA) pathway. Expression of *ORA59* and *PDF1.2* are activated by JA and ethylene (ET) pathways. Treatments are control plants (**C**), rhizobacteria-treated plants (**R**), control plants infested with two neonate larvae of *Mamestra brassicae* (**CM**), or rhizobacteria-treated plants infested with *M. brassicae* (**RM**) for 24 hpi. Transcript levels (mean ± SE) of tested genes were normalized relative to reference genes *EF1α* and *FBOX*, and measured relative to the control plants (*N* = 3–5 replicates, each from a pool of shoots collected from 3 to 5 plates). Different letters over the bars indicate significant differences within a line (one-way ANOVA, LSD *post hoc* test, *P* < 0.05), and letters over horizontal line indicate differences between lines (two-way ANOVA, LSD *post hoc* test, *P* < 0.05). (**E**) Working model of rhizobacterial induction of JA- and ET-regulated genes upon caterpillar feeding. Black arrows represent induction, truncated line represents suppression. Grey lines represent findings from previous studies (Schweizer et al. 2013; Verhage et al. 2011)

To test the hypothesis that rhizobacterial colonization triggered enhanced expression of JA- and ET-regulated genes in Col-0, we evaluated gene expression of the JA-regulated genes *MYC2* and *VSP2* and the JA/ET-regulated genes *ORA59* and *PDF1.2* at 24 h after *M. brassicae* infestation*.* The following treatments were compared: control plants infested with *M. brassicae* (CM), rhizobacteria-treated plants infested with *M. brassicae* (RM), as well as uninfested control plants (C) and rhizobacteria-treated plants (R) in wild type *A. thaliana* Col-0, and the mutants *myc2* and *ora59.* In Col-0 plants, feeding damage by *M. brassicae* on control plants (CM) and rhizobacteria-treated plants (RM) resulted in a slight up-regulation of *MYC2* in comparison to control plants (C), but rhizobacterial colonization did not affect this induction (Fig. 1a). In the mutants *myc2* and *ora59*, the expression of *MYC2* was lower in comparison to its expression in Col-0, and it was not induced by herbivory. The mutants *myc2* and *ora59* are null mutants, the fact that expression of *MYC2* and *ORA59* genes was found in each of the mutants suggests that there are proteins, which have similar functions, thus the expression of the down-stream genes is not 100% reduced. Similar to the expression of *MYC2*, in Col-0, feeding damage by *M. brassicae* on control plants (CM) and rhizobacteria-treated plants (RM) resulted in up-regulation of *VSP2* in comparison to control plants (C), and this up-regulation was not affected by rhizobacterial colonization (Fig. 1b). In the mutants *myc2* and *ora59*, the expression of *VSP2* was comparable to its expression in Col-0, and also was induced by herbivory. Overall, the JA-regulated genes *MYC2* and *VSP2* are induced by herbivory, but are not affected by rhizobacterial colonization.

In *A. thaliana* Col-0 plants, both rhizobacterial colonization with or without the herbivore (R and RM), and herbivory by *M. brassicae* (CM) resulted in up-regulation of *ORA59* (Fig. 1c). The expression of *ORA59* in R plants was higher than in CM and similar to RM. In the mutants *myc2* and *ora59*, the expression of the gene *ORA59* was lower in comparison to its expression in Col-0, and only slightly induced by rhizobacterial colonization (R and RM treatments) although not by herbivory (CM treatment). Similar to the expression pattern of *ORA59*, at 24 hpi in Col-0 plants, rhizobacterial colonization with or without herbivory (R and RM), and *M. brassicae* feeding (CM) resulted in up-regulation of *PDF1.2* (Fig. 1d), although the induction by rhizobacterial colonization was stronger than by herbivory. In the mutant *myc2*, the induction of *PDF1.2* was stronger than in Col-0, and also was induced by herbivory and rhizobacterial colonization. In contrast, in the mutant *ora59*, the induction of *PDF1.2* was lower than in Col-0 plants, and was only slightly induced by rhizobacterial colonization but not by herbivory. Taken together, rhizobacterial colonization resulted in higher levels of expression of the JA/ET-regulated genes *PDF1.2* and *ORA59* than herbivore feeding alone (Fig. 1c-e).

### Rhizobacterial Colonization Enhances the Synthesis of Aliphatic GLS and Suppresses the Herbivore-Induced Levels of Indolic GLS

**Fig 2:**
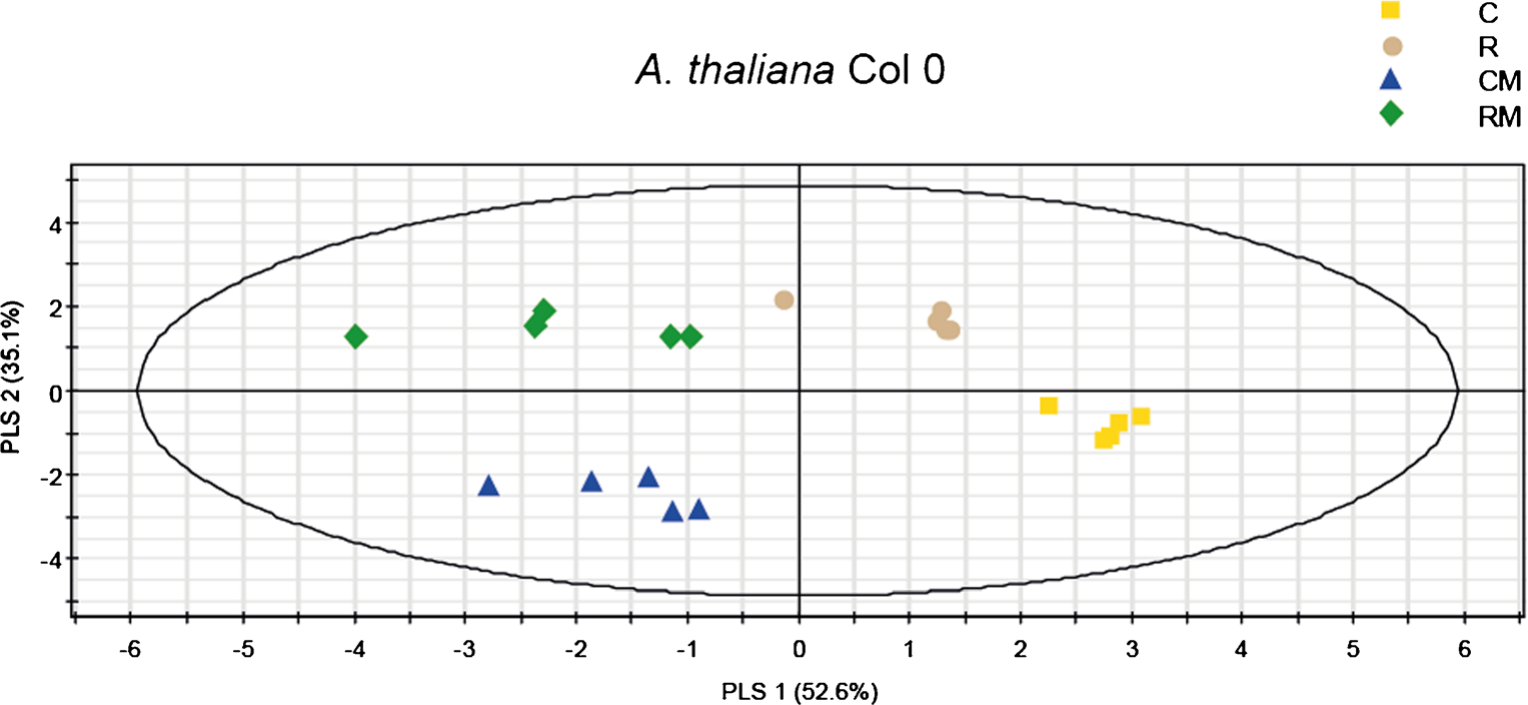
Projection to Latent Structures Discriminant Analysis (PLS-DA) comparison of *Arabidopsis thaliana* Col-0 GLS profiles in shoots of control plants (**C**), rhizobacteria-treated plants (**R**), control plants infested with *Mamestra brassicae* (**CM**), or rhizobacteria-treated plants infested with *M. brassicae* (**RM**). Grouping pattern of samples according to the first two principal components and the Hotelling’s ellipse of the 95% confidence interval for the observations. Each point represents a replicate (*N* = 5), each replicate consisted of a pooled sample of *A. thaliana* shoots collected from 5 plates

The analysis of GLS in *A. thaliana* Col-0 was repeated twice (Exp. 2 and 3) with similar results (Tables S1-S3). The Projection to Latent Structures-Discriminant Analysis (PLS-DA) of data sets from both experiments showed a similar pattern, and here only the PLS-DA plot from experiment 2 is shown (Fig. 2). A PLS-DA analysis of GLS in the four treatments showed two significant principal components (PC) explaining 52.6 and 35.1% of the total variance, respectively. The first PC separated the GLS based on the presence/absence of caterpillars, whereas the second PC separated the GLS based on the presence/absence of rhizobacteria. In the shoot, a total of six aliphatic and four indolic GLS were detected. Three aliphatic GLS and two indolic GLS had a VIP value higher than 1 (Table S3, Exp. 2 and 3). VIP values indicate the variable importance in the projection, and those larger than 1 are the most influential for the model (Eriksson et al. 2006). In decreasing order of importance, these compounds were 8MSOO (glucohirsutin), 7MSOH (glucoibarin), 1MOI3M (neoglucobrassicin), I3M (glucobrassicin), and 4MTB (glucoerucin) (Table S3). In *A. thaliana* Col-0, the aliphatic GLS 3MSOP, 7MSOH, and 8MSOO were induced by rhizobacterial colonization, with the highest levels in RM plants (Tables S1, S2). In contrast, the aliphatic GLS 4MSOB and 5MSOP were induced by herbivory, whereas the aliphatic GLS 4MTB occurred at significantly lower levels in plants exposed to herbivory than in uninfested plants. Interestingly, the indolic GLS 1MOI3M and I3M were induced only by herbivory, with higher levels in CM than in RM. In the mutants *dde2-2*, *ein2-1*, *myc2*, and *ora59*, GLS profiles were separated based on the presence or absence of caterpillars or rhizobacteria in a similar way to Col-0 (Fig. S1).

**Fig 3:**
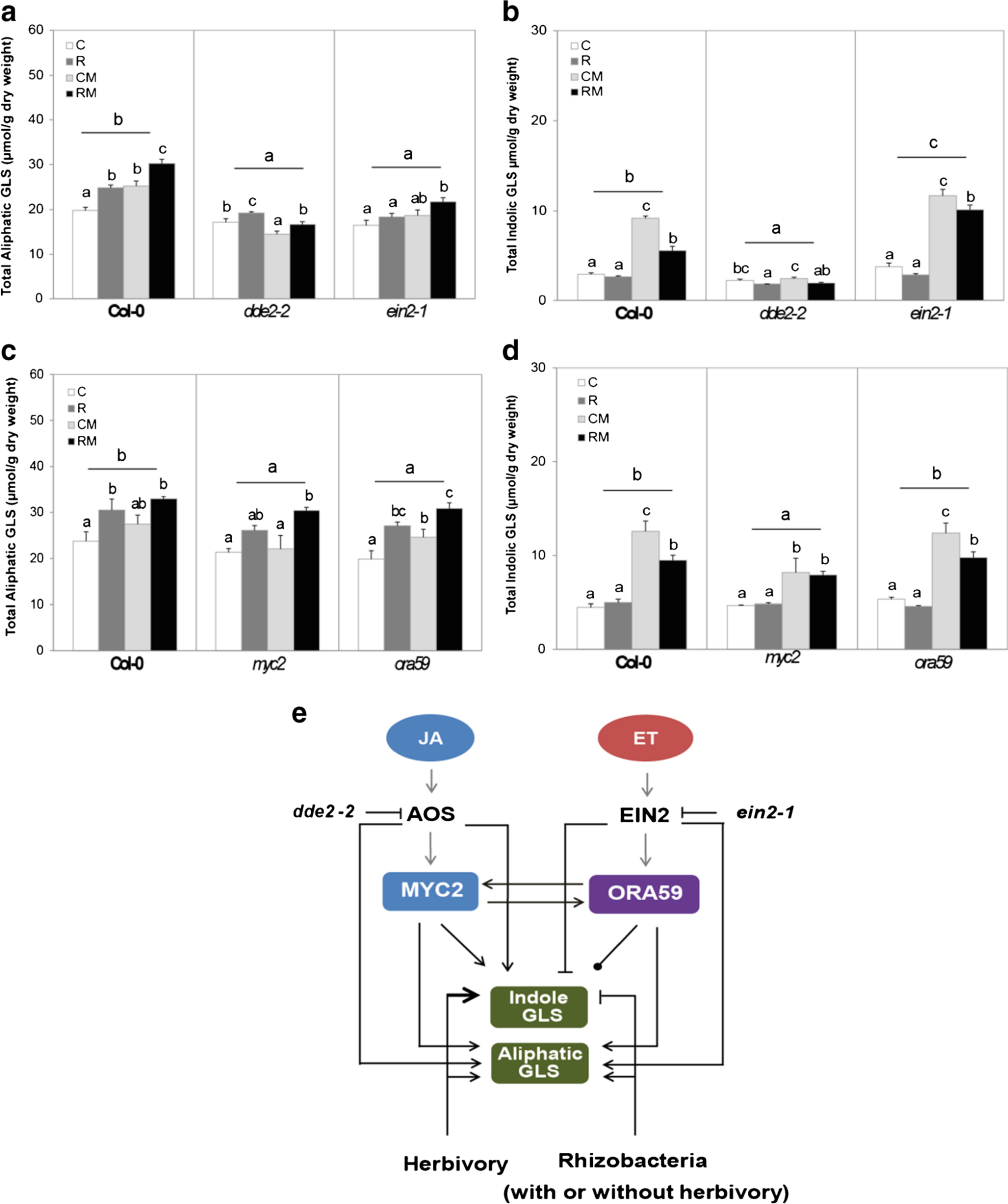
Total levels of aliphatic (**a**, **c**) and indole glucosinolates (**b**, **d**) in shoots of *Arabidopsis thaliana* Col-0, and the mutants *dde2-2*, *ein2-1, myc2,* and *ora59*. Treatments are control plants (**C**), rhizobacteria-treated plants (**R**), control plants infested with two neonate larvae of *Mamestra brassicae* (**CM**), or rhizobacteria-treated plants infested with *M. brassicae* (**RM**) for 4 d (*N* = 5 replicates, each consisting of a pool of shoots collected from 5 plates). Different letters above the bars indicate significant differences within a line (one-way ANOVA, LSD *post hoc* test, *P* < 0.05), and letters above horizontal lines indicate differences between lines (two-way ANOVA, LSD *post hoc* test, *P* < 0.05). Panels A and B, on the one hand, and C and D, on the other, represent two different experiments. (**E**) Working model of rhizobacteria-triggered modification of GLS profile upon caterpillar feeding (RM) represented in black lines compared to control plant infested with caterpillar (CM) in *A. thaliana* represented in grey lines. *Arrows* represent induction, truncated lines represent suppression; line ending in a dot indicates no effect. *Grey arrows* represent information from literature

We evaluated the effect of treatment on GLS levels in wild type *A. thaliana* Col-0, and in the mutants *dde2-2*, *ein2-1*, *myc2,* and *ora59* to test the hypothesis that functional JA- and ET-signaling pathways are required for rhizobacterial modification of the synthesis of GLS. The total levels of aliphatic glucosinolates were reduced in all four mutants compared to the levels in Col-0 (Figs. 3a, c). A first experiment showed that rhizobacterial colonization (R), *M. brassicae* feeding (CM), and the combination of both treatments (RM) induced the biosynthesis of aliphatic GLS in Col-0, with higher levels in RM than in CM (Fig. 3a, Table S1). In contrast to what was observed for Col-0, in mutants *dde2-2* and *ein2-1* herbivory alone did not induce the levels of aliphatic GLS (Fig. 3a)*.* A subsequent experiment with *A. thaliana* Col-0, *myc2,* and *ora59* plants, showed that only rhizobacterial colonization (treatments R and RM) induced the synthesis of aliphatic GLS in Col-0, and such induction was conserved in the *myc2* and *ora59* mutants (Fig. 3c). Taken together, these results show that the biosynthesis of aliphatic GLS requires functional JA- and ET- signaling pathways, and that rhizobacterial colonization induced the levels of aliphatic GLS (Fig. 3e).

In contrast, levels of total indolic GLS were reduced in the JA mutants *dde2-2* and *myc2*, but did not change in *ora59*, and even increased in *ein2-1* compared to Col-0 plants (Figs. 3b, d). In the two experiments with *A. thaliana* Col-0, feeding by *M. brassicae* (CM) induced the synthesis of indolic GLS, but in contrast to the levels of aliphatic glucosinolates, rhizobacterial colonization suppressed the synthesis of the indolic GLS upon caterpillar feeding (RM) (Figs. 3b, d). Interestingly, the suppressive effect of rhizobacteria on herbivore-induced levels of indolic GLS in Col-0, as seen in the comparison between CM and RM, was not observed in the *myc2* mutant, whereas the herbivore-induction of indole GLS was not observed in the *dde2-2* plants. Overall, *M. brassicae* feeding on control plants (CM) induced synthesis of indolic GLS to a higher level compared to feeding on rhizobacteria-colonized plants (RM), and this effect on indolic GLS was mediated by JA signaling (Fig. 3e).

### Rhizobacterial Colonization and Herbivory Induce Biosynthesis of Camalexin

**Fig 4:**
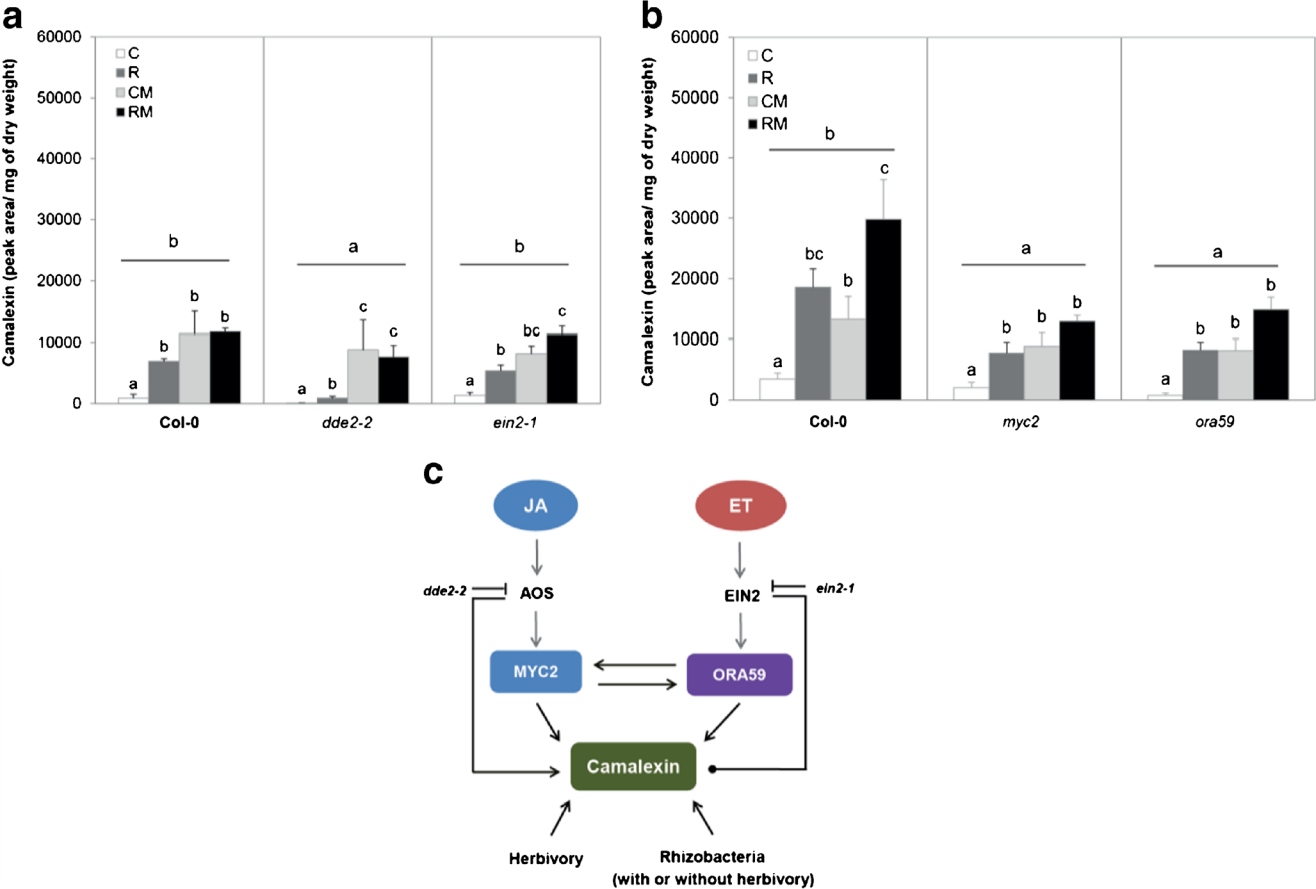
Relative concentration of camalexin (expressed in peak area units) in shoots of **a** *Arabidopsis thaliana* Col-0 and the mutants *dde2-2* and *ein2-1*. **b** *A. thaliana* Col-0 and the mutants *myc2* and *ora59*. Treatments are: control plants **c**, rhizobacteria-treated plants (**R**), control plants infested with *Mamestra brassicae* (**CM**), or rhizobacteria-treated plants infested with two neonate larvae of *M. brassicae* (**RM**) for 4 d (*N* = 5 replicates, each composed of pooled shoots collected from 5 plates). Panels A and B represent two different experiments. Different letters over the bars indicate significant differences within a line (one-way ANOVA, LSD *post hoc* test, *P* < 0.05), and letters over *horizontal line* indicate differences between lines (two-way ANOVA. LSD *post hoc* test, *P* < 0.05). *Arrows* represent induction, *line* ending in a dot indicates that no effect was found

We evaluated camalexin levels, comparing the four treatments in wild type *A. thaliana* Col-0, and in the mutants *dde2-2*, *ein2-1*, *myc2,* and *ora59* to test the hypothesis that functional JA- and ET-signaling pathways are required for rhizobacterial modification of the biosynthesis of this plant defensive compound. The biosynthesis of camalexin was reduced in the *dde2-2, myc2,* and *ora59* mutants compared to Col-0 but not in the *ein2-1* mutants. In Col-0, rhizobacterial colonization (R), *M. brassicae* feeding (CM), and the combination of both treatments (RM) induced camalexin, and this pattern was conserved in the *dde2-2, ein2-1, myc2,* and *ora59* mutants (Fig. 4a-c).

### Rhizobacteria-Mediated Induced Systemic Resistance Against the Generalist Caterpillar *M. brassicae* Requires Functional JA- and ET-Signaling Pathways but is Independent of the Transcription Factors MYC2 and ORA59

**Fig 5:**
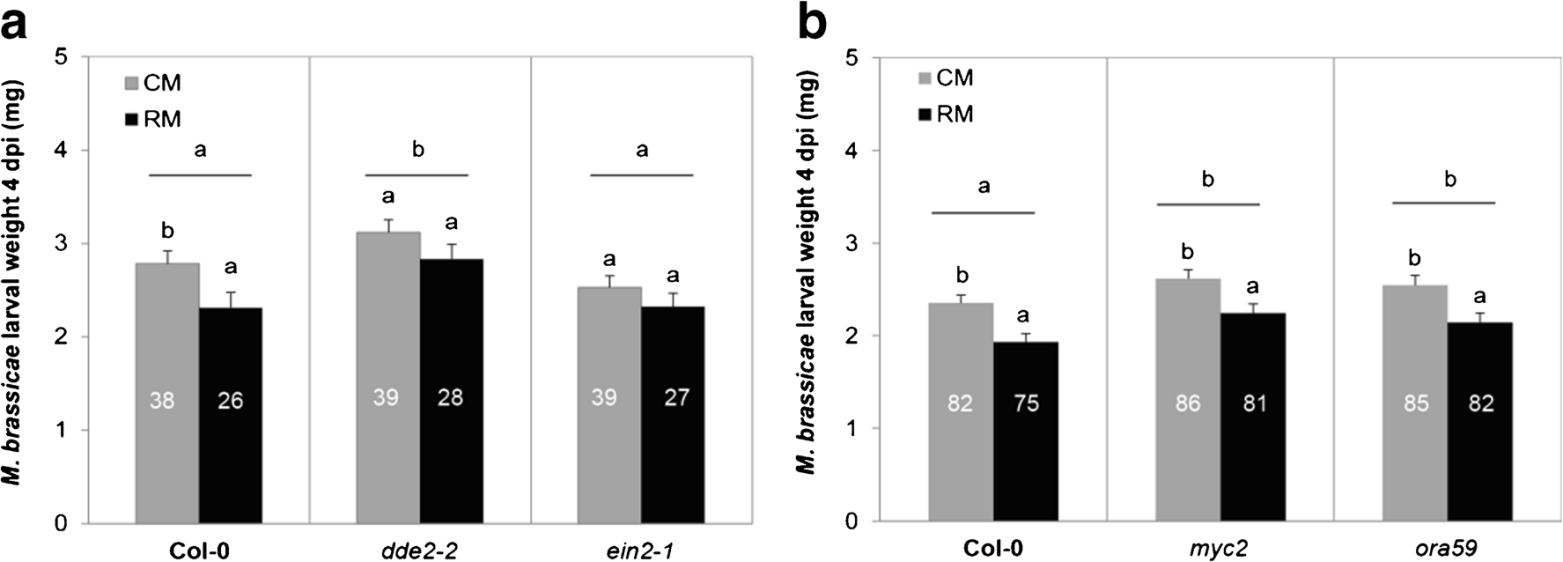
Performance of *Mamestra brassicae* on control (CM) or rhizobacteria-treated plants (RM). Panels **a** (*N* = 14 – 23 plates) and **b** (*N* = 40 – 42 plates) represent two different experiments, and data of panel B are from two independent experiments (see Fig. S1 for extra information). Larval weight was measured at 4 dpi, after infesting each plate with two neonate larvae. Numbers in each bar represent number of larvae surviving on the day of weight measurement. Data shown are means (± SE) of larval weight. Different letters over the bars indicate significant differences within line, and letters over *horizontal line* indicate differences between lines (LMM, *P* < 0.05, LSD test)

We evaluated the performance of *M. brassicae* caterpillars feeding on control (CM) and rhizobacteria-treated (RM) plants for Col-0 wild type and the mutants *dde2-2, myc2, ein2-1*, and *ora59*, to test the hypothesis that rhizobacteria-induced plant resistance to *M. brassicae* requires intact JA- and ET-signaling pathways. In Col-0 plants, rhizobacterial colonization resulted in reduced larval weight of *M. brassicae* (*df* = 1, 31.5; Wald stat. = 4.94; *P* = 0.034). In contrast, when feeding on *dde2-2* and *ein2-1* plants, rhizobacterial colonization did not affect larval weight (Fig. 5a). In a different experiment, rhizobacterial colonization of *A. thaliana* Col-0 consistently resulted in reduced larval weight of *M. brassicae,* both in Col-0 (*df* = 1, 77.4; Wald stat. = 11.81; *P* < 0.001), and when feeding on *myc2* and o*ra59* (Fig. 5b) (*myc2*: *df* = 1, 84.5; Wald stat. = 6.98; *P* = 0.01; *ora59*: *df* = 1, 85.4; Wald stat. = 7.77; *P* = 0.007). Data presented in Fig. 5b are the combined results on *M. brassicae* performance from two independent experiments. In all experiments, the density of rhizobacteria colonizing the roots was above the required threshold for ISR induction (Table S4). Taken together, the rhizobacteria-mediated ISR resulted in reduced *M. brassicae* larval weight compared to control treatments, and whereas functional JA- and ET-signaling were required, the rhizobacteria-mediated ISR was independent of the transcription factors MYC2 and ORA59.

Rhizobacterial colonization had a strong effect on the shoot and root fresh weight in comparison to control plants. Under caterpillar attack, rhizobacteria-treated plants (RM) had stronger shoot and root growth in comparison to control plants infested with caterpillars (CM). In the mutants *dde2-2*, *ein2-1*, *myc2,* and *ora59*, shoot and root biomass of plants in the four treatment groups showed a pattern similar to that of wild type plants (Fig. S2).

## Discussion

The present study shows a consistent effect of the rhizobacterium *P. simiae* WCS417r in triggering ISR that negatively affects the performance of the generalist caterpillar *M. brassicae*. The results show that while herbivory by this generalist herbivore prioritizes activation of the MYC2-branch of the JA-signaling pathway in the plant, rhizobacterial colonization causes a shift to a stronger activation of the ORA59-branch of the JA-signaling pathway (Fig. 1). However, the transcription factor ORA59 is not the only responsible transcription factor for the observed effect of rhizobacterial colonization on caterpillar growth, based on the observation that the effect of rhizobacterial colonization on *M. brassicae* larval weight remained the same on the *ora59* mutant. Instead, we observed that functional JA- and ET-signaling pathways are required for rhizobacteria-ISR against *M. brassicae.* The rhizobacterium *P. simiae* WCS417r (formerly *P. fluorescens* WCS417r) used in this study is known to induce ISR against pathogens *via* the JA/ET signaling pathways too (Pieterse et al. 2002; 2014; Van der Ent et al. 2009). This suggests similarity in the mechanism by which plants mount an effective response upon rhizobacterial colonization against different types of attackers.

Rhizobacterial colonization alone or in combination with infestation by *M. brassicae* increased the expression of both the JA/ET-regulated transcription factor ORA59 and the marker gene *PDF1.2*. Using the same rhizobacterium-plant combination, previous studies found that rhizobacterial colonization of plant roots enhanced expression of *PDF1.2* only after herbivory (Pangesti et al. 2015; Pineda et al. 2012; Van Oosten et al. 2008), a phenomenon known as “priming” of induced plant defense (Conrath 2009). Interestingly, our results show that rhizobacterial colonization alone (R) induced expression of *PDF1.2* to the same levels as in the combined treatment of rhizobacterial colonization and *M. brassicae* infestation (RM). The gene *PDF1.2* encodes a plant defensin that is a basic peptide having antimicrobial activity against pathogens (Sels et al. 2008; Thomma et al. 2002). Here, we show that colonization by the beneficial rhizobacterium *P. simiae* WCS417r also induces the expression of *PDF1.2*, which suggests that the plant initially recognizes this rhizobacterium as a pathogenic organism, and therefore, expresses defense-associated genes that limit its colonization. The results support a new interesting aspect of beneficial microbe-plant interactions, as it has been proposed that plants initially recognize the root-associated microbes as attacker, and therefore, produce compounds that can limit the development of microbes (Pozo and Azcon-Aguilar 2007; Zamioudis and Pieterse 2012). Another hypothesis is that beneficial microbes induce plant defensive compounds that could limit its competitors for plant resources. The role of defensin peptides in the inhibition of bacterial and fungal growth has been extensively reported, whereas in plant defense to herbivorous insects, few studies have addressed their role. An expression of BrD1 protein, a plant defensin from *Brassica rapa*, in transgenic rice has suggested that the protein has insecticidal activity to nymphs of the brown planthopper *Nilaparvata lugens* (Choi et al. 2009). In a study on *A. thaliana*, it was found that *P. rapae* oral secretion induces the expression of *PDF1.2* (Verhage et al. 2011). Interestingly, there is a strong interest in the importance of microbes in insect oral secretions or gut for the immune system and growth of herbivores as reviewed in Engel and Moran (2013), that can also influence plant-insect interactions. Further studies are required to investigate if the up-regulation of defensin peptides in plants colonized by rhizobacteria could negatively affect insect-associated microbes and suppress the insect’s immune system, thus making the plant more resistant to the insect herbivore.

Our data show that rhizobacterial colonization consistently enhanced the levels of aliphatic GLS, suppressed the herbivore-induced levels of indolic GLS, and thereby significantly affected GLS composition. Moreover, rhizobacterial colonization or caterpillar feeding also consistently induced synthesis of camalexin in the shoot. Using the same method of *in vitro* assays, colonization of *A. thaliana* Col-0 roots by the *P. fluorescens* strain SS101 induced accumulation of both aliphatic and indolic GLS in the shoots and in the roots, in the absence of herbivory (Van de Mortel et al. 2012), as well as upregulation of camalexin synthesis in local and systemic tissues. Our present study and the study by Van de Mortel et al. (2012) indicate that different strains of a rhizobacterial species colonizing *Arabidopsis* roots can influence the chemical composition in systemic tissues that may contribute to ISR to herbivorous insects. Previous studies mostly have indicated that camalexin is regulated *via* the SA pathway (Glawischnig 2007; Glazebrook 2005). The results of the present study show that the JA pathway and transcription factors MYC2 and ORA59 also are involved in the regulation of camalexin synthesis. Our results, thus, reveal new aspects of the biological role of camalexin and the role of signaling pathways underlying the synthesis of this compound. The experiments with mutants, as presented here, show that activation of the JA signaling pathway induces the synthesis of aliphatic and indolic GLS, whereas the ET pathway represses the synthesis of indolic GLS. By modulating both the JA- and ET pathways, *P. simiae* WCS417r colonization, changes GLS composition by enhancing the biosynthesis of aliphatic GLS and suppressing the biosynthesis of indolic GLS that is induced by herbivory. The sensitivity of *M. brassicae* to aliphatic GLS has been reported in a previous study (Beekwilder et al. 2008), showing that in the *A. thaliana myb28myb29* double mutant lacking aliphatic GLS, the weight of *M. brassicae* increased 2.6 fold compared to the performance in wild type Col-0. Moreover, a negative correlation between the concentration of aliphatic GLS and performance of the generalist caterpillar *S. exigua* and the specialist *P. rapae* has been reported (Kos et al. 2012). Taken together, we propose that accumulation of aliphatic GLS synthesis can underly *P. simiae* WCS417r-mediated ISR against leaf-chewing *M. brassicae* caterpillars. A study using mutants lacking aliphatic GLS may unravel the underlying mechanisms of rhizobacteria-plant-insect interactions.

By using a closed *in vitro* system, we here showed that colonization by the rhizobacterium *P. simiae* WCS417r had a consistent negative effect on the performance of the generalist caterpillar *M. brassicae*, and that this is associated with prioritization of the JA/ET-regulated ORA59-branch over the JA-regulated MYC2-branch in the presence and absence of herbivory. By using an open system in soil, we previously found that the effect of rhizobacterium *P. simiae* WCS417r on plant direct defense against *M. brassicae* was variable, depending on soil composition (Pangesti et al. 2015). Moreover, it is known that in response to microbial attack and herbivore feeding, plants produce high levels of ET (De Vos et al. 2005), although microbial attack induces higher levels of ET in comparison to herbivore feeding. In this study, experiments were conducted in a closed system, and it is possible that in the early stage of rhizobacterial colonization, plants emit ET, and that accumulation of ET triggers prioritization of ORA59-branch even without caterpillar infestation. First, we propose that the prioritization of the ET signaling pathway, enhancing the synthesis of aliphatic GLS even without herbivore attack, may strengthen rhizobacteria-mediated ISR against the caterpillar *M. brassicae*. However, a mutation in the JA/ET-regulated *ORA59*, which is downstream of the knock-out genes in *dde2-2* and *ein2-1* mutants, does not have any effect on induction of rhizobacteria-mediated ISR against the caterpillars, suggesting that ORA59 alone does not explain rhizobacterial induced ISR against *M. brassicae*. It is possible that there is functional redundancy of ORA59 provided by other JA- and ET-regulated proteins, thus mutation of only *ORA59* does not change the induction of rhizobacteria-mediated ISR against *M. brassicae*. A similar reasoning may apply for *MYC2*, the function of which may be complemented by MYC3/4 (Schweizer et al. 2013). Since rhizobacteria-mediated ISR against caterpillars required both the JA- and ET signaling pathways (as observed with the mutants *dde2-2* and *ein2-1*), whether other JA-/ET-targeted transcription factors mediate the regulation of rhizobacteria-plant-insect interactions remains to be investigated. Second, we propose that simpler nutrient composition in the half-strength Murashige & Skoog (MS) media used in this study, compared to the nutrients available in soil, may result in more intense interactions between rhizobacteria and plants (Pangesti et al. 2015), and could be one of the factors that contributed to the consistent effect of rhizobacteria-mediated ISR against the generalist caterpillar *M. brassicae*. Moreover, in an open system, other microbes also can colonize plant roots and compete with the rhizobacterium, and may therefore modify the rhizobacterium-plant interactions (Shavit et al. 2013), which is not the case in the *in vitro* system used here.

**Fig 6:**
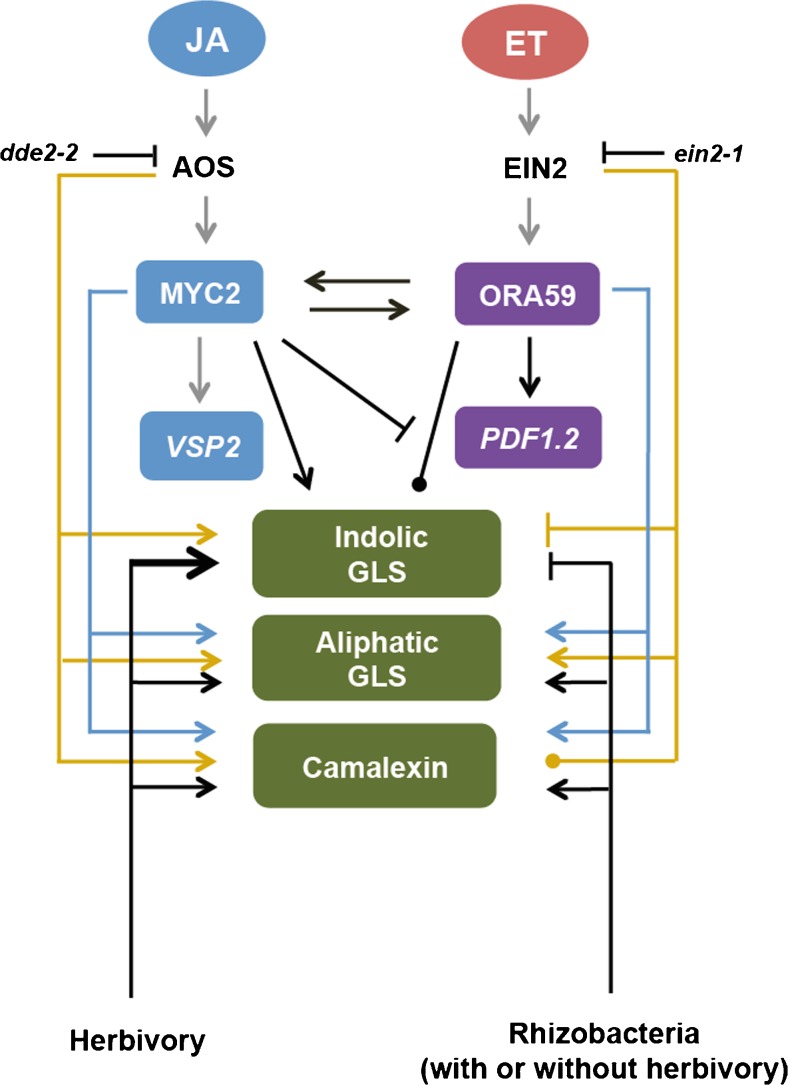
Working model of rhizobacteria-mediated induced systemic resistance (ISR) *via* jasmonic acid/ethylene (JA/ET)-dependent mechanisms based on studies in *Arabidopsis thaliana* Col-0. Rhizobacterial- and herbivory-induced modification of plant gene transcription, glucosinolates and camalexin biosynthesis are represented in *black lines*. Central component of JA- and ET-signaling pathways regulation of plant gene transcription and chemical biosynthesis are represented in orange and blue lines. Grey lines represent results from the literature (Schweizer et al. 2013.; Verhage et al. 2011). *Arrows* represent induction, truncated lines represent suppression; *dotted line* indicates no effect

In the present study, we found evidence that colonization of plant roots by rhizobacteria alters plant-insect interactions at the level of gene transcription, plant chemistry, and insect performance. Previous studies using the same rhizobacterium-plant combination recorded an up-regulation of the JA/ET-regulated genes such as *PDF1.2*, and *HEL* upon feeding by the caterpillars *Spodoptera exigua* (Van Oosten et al. 2008), *M. brassicae* (Pangesti et al. 2015), and the aphid *Myzus persicae* (Pineda et al. 2012). This study furthers our understanding of mechanisms of rhizobacteria-mediated ISR against leaf-chewing insects by showing not only that functional JA- and ET pathways are required, but also that ISR against *M. brassicae* is induced along the ORA59 branch and not the MYC2 branch of the octadecanoid signal-transduction pathway. Furthermore, this study also provides new information on the induction of defensive compounds, such as glucosinolates and camalexin by rhizobacterial colonization that can potentially explain the increased resistance to insect herbivores (Fig. 6). Recent experimental evidence is uncovering the beneficial contribution of microorganisms to the functioning of humans, insects, and plants by affecting growth, development, and immunity to diseases (Engel and Moran 2013; Selosse et al. 2014; Sugio et al. 2014). In rhizobacteria-colonized plants, we found a higher expression of the *PDF1.2* gene that encodes a plant defensin peptide, and higher levels of secondary metabolites such as camalexin, both known to have antimicrobial activity. This may be an initial response of plants to the recognition of the beneficial microbes. Whether these compounds have activity beyond antimicrobial effects, thus directly influencing insect physiology, or whether the compounds affect the insects indirectly by changing insect-associated microbes, thus modifying plant-insect interactions, could be fruitful questions for future research to unveil the mechanisms underlying beneficial microbe-plant-insect interactions.

## Electronic supplementary material

Below is the link to the electronic supplementary material.

ESM 1 (DOCX 316 kb)
