# Supplementary material for: Jasmonic Acid and Ethylene Signaling Pathways Regulate Glucosinolate Levels in Plants During Rhizobacteria-Induced Systemic Resistance Against a Leaf-Chewing Herbivore
Source: J Chem Ecol. 2016 Nov 15;42(12):1212–25. doi: 10.1007/s10886-016-0787-7 (PMC5148788; doi:10.1007/s10886-016-0787-7)
Supplement: Supplementary file 1 — (DOCX 316 kb) [file 10886_2016_787_MOESM1_ESM.docx]

**Supplementary Data**

JASMONIC ACID AND ETHYLENE SIGNALING PATHWAYS REGULATE GLUCOSINOLATE LEVELS IN PLANTS DURING RHIZOBACTERIA-INDUCED SYSTEMIC RESISTANCE AGAINST A LEAF-CHEWING HERBIVORE

NURMI PANGESTI^1*^, MICHAEL REICHELT^2^, JUDITH E. VAN DE MORTEL^3,4^, ELENI KAPSOMENOU^1^, JONATHAN GERSHENZON^2^, JOOP J.A. VAN LOON^1^, MARCEL DICKE^1^, and ANA PINEDA^1,5^

*^1^ Wageningen University, Laboratory of Entomology, P.O. Box 16, 6700 AA Wageningen, The Netherlands*

*^2^Max Planck Institute for Chemical Ecology, Department of Biochemistry, 07745 Jena, Germany*

*^3^ Wageningen University, Laboratory of Phytopathology, P.O. Box 16, 6700 AA Wageningen, The Netherlands*

*^4^Current address: HAS University of Applied Sciences, 5911 KJ Venlo, The Netherlands.*

*^5^Current address: Department of Terrestrial Ecology, Netherlands Institute of Ecology (NIOO-KNAW), PO Box 50, 6700 AB, Wageningen, The Netherlands*

^*^Corresponding author: [npangesti001@gmail.com](mailto:npangesti001@gmail.com)

Running title: Plant responses to rhizobacteria and herbivores

**Table S1** Contents of aliphatic and indolic glucosinolates (in µmol/g dry weight) in the shoot of *Arabidopsis thaliana* ecotype Col-0, jasmonic (JA)-biosynthesis mutant *dde2-2* and ethylene-insensitive mutant *ein2-1*, in control plants **(C)**, rhizobacteria-treated plants **(R)**, control plants infested with *Mamestra brassicae* **(CM)**, or rhizobacteria-treated plants infested with *M. brassicae* **(RM)**.

|  |  | Col 0 | |  |  | *dde2-2* | |  |  | *ein2-1* | |  |
| --- | --- | --- | --- | --- | --- | --- | --- | --- | --- | --- | --- | --- |
|  | **C** | **R** | **CM** | **RM** | **C** | **R** | **CM** | **RM** | **C** | **R** | **CM** | **RM** |
|  |  |  |  |  |  |  |  |  |  |  |  |  |
| Aliphatic |  |  |  |  |  |  |  |  |  |  |  |  |
| 3MSOP | 1.91 ± 0.11  **a** | 2.54 ± 0.06  **b** | 2.66 ± 0.17  **b** | 3.27 ± 0.07  **c** | 1.57 ± 0.11  **a** | 2.05 ± 0.22  **b** | 2.00 ± 0.06  **b** | 1.51 ± 0.10  **a** | 1.52 ± 0.11  **a** | 1.79 ± 0.09  **ab** | 2.01 ± 0.18  **b** | 2.14 ± 0.09  **b** |
| 4MSOB | 15.80 ± 0.56  **a** | 17.17 ± 0.51  **a** | 20.94 ± 0.82  **b** | 21.67 ± 0.98  **b** | 12.61 ± 0.72  **a** | 12.58 ± 0.10  **a** | 10.86 ± 0.71  **a** | 11.65 ± 0.57  **a** | 12.78 ± 0.83  **a** | 12.63 ± 0.62  **a** | 15.20 ± 0.96  **b** | 15.42 ± 2.00  **b** |
| 7MSOH | 0.54 ± 0.02  **a** | 1.11 ± 0.05  **b** | 0.56 ± 0.03  **a** | 1.23 ± 0.06  **c** | 0.66 ± 0.03  **b** | 0.95 ± 0.02  **d** | 0.55 ± 0.02  **a** | 0.81 ± 0.02  **c** | 0.53 ± 0.05  **a** | 0.94 ± 0.06  **b** | 0.62 ± 0.03  **a** | 1.24 ± 0.07  **c** |
| 4MTB | 0.78 ± 0.08  **b** | 0.63 ± 0.07  **b** | 0.06 ± 0.02  **a** | 0.17 ± 0.04  **a** | 1.38 ± 0.15  **b** | 1.16 ± 0.11  **b** | 0.48 ± 0.12  **a** | 0.67 ± 0.07  **a** | 1.05 ± 0.17  **b** | 1.03 ± 0.11  **b** | 0.15 ± 0.02  **a** | 0.11 ± 0.01  **a** |
| 8MSOO | 0.81 ± 0.05  **a** | 3.38 ± 0.13  **b** | 1.00 ± 0.07  **a** | 3.88 ± 0.15  **c** | 0.97 ± 0.08  **b** | 2.52 ± 0.11  **d** | 0.65 ± 0.06  **a** | 2.02 ± 0.03  **c** | 0.60 ± 0.05  **a** | 1.98 ± 0.19  **b** | 0.70 ± 0.11  **a** | 2.78 ± 0.20  **c** |
| Sub total | 19.84 ± 0.70  **a** | 24.84 ± 0.60  **b** | 25.23 ± 1.05  **b** | 30.22 ± 0.95  **c** | 17.20 ± 0.78  **b** | 19.27 ± 0.28  **c** | 14.54 ± 0.66  **a** | 16.67 ± 0.64  **b** | 16.48 ± 1.15  **a** | 18.36 ± 0.82  **a** | 18.68 ± 1.26  **ab** | 21.70 ± 0.89  **b** |
|  |  |  |  |  |  |  |  |  |  |  |  |  |
| Indolic |  |  |  |  |  |  |  |  |  |  |  |  |
| 4OHI3M | 0.24 ± 0.01  **a** | 0.30 ± 0.02  **b** | 0.29 ± 0.01  **ab** | 0.36 ± 0.03  **c** | 0.16 ± 0.01  **a** | 0.17 ± 0.01  **a** | 0.16 ± 0.02  **a** | 0.17 ± 0.00  **a** | 0.18 ± 0.01  **a** | 0.21 ± 0.01  **ab** | 0.24 ± 0.01  **b** | 0.32 ± 0.01  **c** |
| I3M | 1.07 ± 0.08  **b** | 0.66 ± 0.02  **a** | 1.94 ± 0.11  **c** | 1.11 ± 0.09  **b** | - 1. ± 0.08   **b** | 0.57 ± 0.01  **a** | 0.88 ± 0.08  **b** | 0.52 ± 0.04  **a** | 1.41 ± 0.13  **b** | 0.84 ± 0.05  **a** | 2.36 ± 0.11  **c** | 2.09 ± 0.15  **c** |
| 4MOI3M | 0.25 ± 0.02  **a** | 0.26 ± 0.02  **ab** | 0.33 ± 0.00  **c** | 0.31 ± 0.03  **bc** | 0.27 ± 0.03  **a** | 0.24 ± 0.03  **a** | 0.29 ± 0.04  **a** | 0.23 ± 0.02  **a** | 0.26 ± 0.03  **b** | 0.27 ± 0.01  **b** | 0.12 ± 0.01  **a** | 0.30 ± 0.03  **b** |
| 1MOI3M | 1.39 ± 0.07  **a** | 1.45 ± 0.08  **a** | 6.62 ± 0.20  **c** | 3.79 ± 0.38  **b** | 0.81 ± 0.08  **a** | 0.87 ± 0.03  **ab** | 1.13 ± 0.07  **c** | 1.05 ± 0.08  **bc** | 1.94 ± 0.28  **a** | 1.57 ± 0.14  **a** | 8.96 ± 0.62  **c** | 7.43 ± 0.38  **b** |
| Sub total | 2.95 ± 0.15  **a** | 2.67 ± 0.11  **a** | 9.19 ± 0.25  **c** | 5.57 ± 0.50  **b** | 2.25 ± 0.15  **bc** | 1.86 ± 0.02  **a** | 2.46 ± 0.16  **c** | 1.97 ± 0.08  **ab** | 3.79 ± 0.40  **a** | 2.88 ± 0.13  **a** | 11.67 ± 0.71  **c** | 10.14 ± 0.51  **b** |
|  |  |  |  |  |  |  |  |  |  |  |  |  |
| Total | 22.79 ± 0.71  **a** | 27.51 ± 0.59  **b** | 34.42 ± 1.20  **c** | 35.79 ± 1.36  **c** | 19.45 ± 0.82  **bc** | 21.12 ± 0.29  **c** | 17.00 ± 0.61  **a** | 18.63 ± 0.64  **ab** | 20.27 ± 1.52  **a** | 21.24 ± 0.73  **a** | 30.35 ± 1.87  **b** | 31.83 ± 1.10  **b** |

**Aliphatic GLS:** 3MSOP (glucoiberin), 4MSOB (glucoraphanin), 7MSOH (glucoibarin), 4MTB (glucoerucin), 8MSOO (glucohirsutin). **Indolic GLS:** 4OHI3M (4-hydroxy-glucobrassicin), I3M (glucobrassicin), 4MOI3M (4-methoxy-glucobrassicin), 1MOI3M (neoglucobrassicin).

**Table S2** Contents of aliphatic and indole glucosinolates (µmol/g dry weight) in the shoot of *Arabidopsis thaliana* ecotype Col-0 and jasmonic acid (JA)-regulated transcription factor mutant *myc2* and JA/ET-regulated transcription factor mutant *ora59* in control plants **(C)**, rhizobacteria-treated plants **(R)**, control plants infested with *Mamestra brassicae* **(CM)**, or rhizobacteria-treated plants infested with *M. brassicae* **(RM)**.

|  |  | Col-0 | |  |  | *myc2* | |  |  | *ora59* | |  |
| --- | --- | --- | --- | --- | --- | --- | --- | --- | --- | --- | --- | --- |
|  | **C** | **R** | **CM** | **RM** | **C** | **R** | **CM** | **RM** | **C** | **R** | **CM** | **RM** |
| Aliphatic |  |  |  |  |  |  |  |  |  |  |  |  |
| 3MSOP | 1.89 ± 0.19 **a** | 2.56 ± 0.19  **bc** | 2.15 ± 0.17  **ab** | 2.71 ± 0.09  **c** | 1.55 ± 0.09  **a** | 2.21 ± 0.10  **b** | 1.76 ± 0.26  **ab** | 2.72 ± 0.08  **c** | 1.45 ± 0.20  **a** | 2.32 ± 0.08  **bc** | 1.95 ± 0.16  **b** | 2.63 ± 0.12  **c** |
| 4MSOB | 12.84 ± 0.86  **a** | 15.70 ± 1.26  **a** | 18.89 ± 1.11  **b** | 19.30 ± 0.51  **b** | 9.92 ± 0.82  **a** | 11.89 ± 0.78  **ab** | 14.72 ± 1.75  **bc** | 16.97 ± 0.59  **c** | 9.14 ± 1.28  **a** | 11.99 ± 0.58  **b** | 15.83 ± 1.14  **c** | 17.36 ± 0.49  **c** |
| 5MSOP | 0.95 ± 0.02  **a** | 1.13 ± 0.10  **a** | 1.42 ± 0.06  **b** | 1.39 ± 0.04  **b** | 0.81 ± 0.09  **a** | 0.80 ± 0.04  **a** | 1.08 ± 0.08  **b** | 1.07 ± 0.05  **b** | 0.69 ± 0.07  **a** | 0.83 ± 0.04  **a** | 1.24 ± 0.05  **b** | 1.25 ± 0.04  **b** |
| 7MSOH | 0.29 ± 0.02  **a** | 0.71 ± 0.07  **b** | 0.37 ± 0.02  **a** | 0.86 ± 0.02  **c** | 0.26 ± 0.01  **a** | 0.44 ± 0.03  **b** | 0.30 ± 0.03  **a** | 0.53 ± 0.01  **c** | 0.27 ± 0.01  **a** | 0.68 ± 0.08  **b** | 0.41 ± 0.01  **a** | 0.80 ± 0.05  **b** |
| 4MTB | 7.13 ± 0.98  **bc** | 7.92 ± 0.76  **c** | 3.72 ± 0.64  **a** | 5.57 ± 0.28  **ab** | 8.24 ± 0.63  **bc** | 9.17 ± 0.32  **c** | 3.69 ± 0.83  **a** | 7.39 ± 0.15  **b** | 7.69 ± 0.53  bc | 8.87 ± 0.14  c | 4.20 ± 0.59  a | 6.30 ± 0.49  b |
| 8MSOO | 0.65 ± 0.05  **a** | 2.49 ± 0.24  **b** | 0.90 ± 0.07  **a** | 3.13 ± 0.12  **c** | 0.58 ± 0.03  **a** | 1.57 ± 0.16  **b** | 0.57 ± 0.09  **a** | 1.75 ± 0.06  **b** | 0.67 ± 0.05  **a** | 2.38 ± 0.41  **b** | 0.96 ± 0.04  **a** | 2.52 ± 0.27  **b** |
| Sub total | 23.76 ± 2.01  **a** | 30.52 ± 2.41  **b** | 27.46 ± 1.96  **ab** | 32.97 ± 0.53  **b** | 21.37 ± 0.82  **a** | 26.09 ± 1.08  **ab** | 22.11 ± 2.89  **a** | 30.41 ± 0.70  **b** | 19.91 ± 1.78  **a** | 27.07 ± 0.82  **bc** | 24.59 ± 1.75  **b** | 30.85 ± 1.25  **c** |
| Indolic |  |  |  |  |  |  |  |  |  |  |  |  |
| 4OHI3M | 0.07 ± 0.01  **ab** | 0.05 ± 0.01  **a** | 0.14 ± 0.01  **c** | 0.10 ± 0.03  **bc** | 0.07 ± 0.01  **a** | 0.09 ± 0.00  **a** | 0.08 ± 0.01  **a** | 0.09 ± 0.01  **a** | 0.02 ± 0.01  **a** | 0.03 ± 0.00  **a** | 0.04 ± 0.00  **a** | 0.04 ± 0.01  **a** |
| I3M | 1.89 ± 0.15  **a** | 1.84 ± 0.09  **a** | 4.07 ± 0.31  **c** | 3.12 ± 0.19  **b** | 1.86 ± 0.02  **a** | 1.48 ± 0.06  **a** | 3.39 ± 0.65  **b** | 2.89 ± 0.07  **b** | 1.95 ± 0.04  **b** | 1.51 ± 0.04  **a** | 3.61 ± 0.23  **d** | 2.81 ± 0.12  **c** |
| 4MOI3M | 1.19 ± 0.03  **a** | 1.34 ± 0.06  **a** | 1.12 ± 0.08  **a** | 1.40 ± 0.12  **a** | 1.19 ± 0.03  **a** | 1.20 ± 0.02  **a** | 1.20 ± 0.09  **a** | 1.35 ± 0.02  **a** | 0.89 ± 0.06  **a** | 0.85 ± 0.02  **a** | 0.83 ± 0.01  **a** | 0.83 ± 0.09  **a** |
| 1MOI3M | 1.34 ± 0.24  **a** | 1.76 ± 0.23  **a** | 7.23 ± 0.81  **c** | 4.86 ± 0.31  **b** | 1.55 ± 0.04  **a** | 2.07 ± 0.11  **a** | 3.53 ± 0.77  **b** | 3.58 ± 0.31  **b** | 2.48 ± 0.25  **a** | 2.21 ± 0.10  **a** | 7.91 ± 0.88  **c** | 6.10 ± 0.46  **b** |
| Sub total | 4.49 ± 0.36  **a** | 4.99 ± 0.35  **a** | 12.56 ± 1.10  **c** | 9.49 ± 0.55  **b** | 4.67 ± 0.06  **a** | 4.83 ± 0.12  **a** | 8.20 ± 1.52  **b** | 7.92 ± 0.38  **b** | 5.34 ± 0.22  **a** | 4.60 ± 0.08  **a** | 12.38 ± 1.08  **c** | 9.77 ± 0.63  **b** |
| TOTAL | 28.25 ± 2.33  **a** | 35.51 ± 2.33  **b** | 40.02 ± 2.88  **bc** | 42.46 ± 0.66  **c** | 26.04 ± 0.85  **a** | 30.92 ± 1.14  **a** | 30.31 ± 4.37  **a** | 38.33 ± 0.60  **b** | 25.25 ± 1.99  **a** | 31.67 ± 0.87  **b** | 36.97 ± 1.85  **c** | 40.62 ± 1.73  **c** |

**Aliphatic GLS:** 3MSOP (glucoiberin), 4MSOB (glucoraphanin), 5MSOP (glucoalyssin), 7MSOH (glucoibarin), 4MTB (glucoerucin), 8MSOO (glucohirsutin). **Indolic GLS:** 4OHI3M (4-hydroxy-glucobrassicin), I3M (glucobrassicin), 4MOI3M (4-methoxy-glucobrassicin), 1MOI3M (neoglucobrassicin).

**Table S3** Variable Importance in the Projection (VIP) values of each glucosinolate compound in the shoot of different *Arabidopsis thaliana* lines. The VIP values relate to Projection to Latent Structures-Discriminant Analysis (PLS-DA)

| **Compound** | **Experiment 2** | | | **Experiment 3** | | |
| --- | --- | --- | --- | --- | --- | --- |
|  | **Col-0** | ***dde2-2*** | ***ein2-1*** | **Col-0** | ***myc2*** | ***ora59*** |
| **Aliphatic** |  | | |  | | |
| 3MSOP | 0.918 | **1.164** | 0.850 | 0.960 | 0.789 | **1.059** |
| 4MSOB | 0.917 | 0.812 | 0.686 | 0.931 | 0.802 | 0.940 |
| 5MSOP | - | - | - | 0.943 | 0.923 | 0.996 |
| 7MSOH | **1.132** | **1.108** | **1.050** | **1.202** | 0.980 | **1.200** |
| 4MTB | 0.937 | **1.208** | 0.855 | **1.026** | **1.316** | **1.063** |
| 8MSOO | **1.178** | **1.098** | **1.103** | **1.236** | **1.066** | **1.154** |
| **Indole** |  |  |  |  |  |  |
| 4OHI3M | 0.911 | 0.475 | 0.873 | 0.806 | **1.068** | 0.702 |
| I3M | **1.064** | **1.135** | **1.123** | **1.024** | **1.026** | **1.107** |
| 4MOI3M | 0.740 | 0.780 | **1.367** | 0.720 | 0.993 | 0.571 |
| 1MOI3M | **1.121** | 0.986 | 0.930 | **1.039** | 0.935 | **1.029** |

- VIP value > 1 written in bold

| **Table S4** Rhizobacterial colonization levels in roots of different plant lines   \| **Experiment** \| **Plant** \| **Replicates** \| **Colony forming unit (CFU)**  **mg^-1^ of roots** \| \| --- \| --- \| --- \| --- \| \| 1 \| Col-0 \| 2 \| 1.18*10^6^ \| \| *myc2* \| 5 \| 4.85*10^5^ \| \| *ora59* \| 4 \| 6.24*10^5^ \| \| 2 \| Col-0 \| 6 \| 2.65*10^5^ \| \| *dde2-2* \| 6 \| 4.38*10^5^ \| \| *ein2-1* \| 6 \| 3.56*10^5^ \| \| 3 \| Col-0 \| 5 \| 1.36*10^5^ \| \| *myc2* \| 5 \| 1.14*10^5^ \| \| *ora59* \| 5 \| 1.00*10^5^ \| |
| --- | --- | --- | --- | --- | --- | --- | --- | --- | --- | --- | --- | --- | --- | --- | --- | --- | --- | --- | --- | --- | --- | --- | --- | --- | --- | --- | --- | --- | --- | --- | --- | --- | --- | --- |

| **Table S5** Sequences of *Arabidopsis thaliana*-derived primers used in quantitative RT-PCR analyses.   \| **Gene** \| **Gene ID** \|  \| **Sequence** \| \| --- \| --- \| --- \| --- \| \| *EF1α* \| At5g60390 \| F  R \| TGAGCACGCTCTTCTTGCTTTCA  GGTGGTGGCATCCATCTTGTTACA \| \| *FBOX* \| At5g15710 \| F  R \| TTTCGGCTGAGAGGTTCGAGT  GATTCCAAGACGTAAAGCAGATCAA \| \| *MYC2* \| At1g32640 \| F  R \| ATCCAAGTTCTTATTCGGGTC  CGTCTTTGTCTCTCTGCTTCG \| \| *ORA59* \| At1g06160 \| F  R \| TTCCCCGGAGAACTCTTCTT  GCCTGATCATAAGCGAGAGC \| \| *PDF1.2* \| At5g44420 \| F  R \| CACCCTTATCTTCGCTGCTC  GTTGCATGATCCATGTTTGG \| \| *VSP2* \| At5g24770 \| F  R \| TCAGTGACCGTTGGAAGTTGTG  GTTCGAACCATTAGGCTTCAATATG \| |
| --- | --- | --- | --- | --- | --- | --- | --- | --- | --- | --- | --- | --- | --- | --- | --- | --- | --- | --- | --- | --- | --- | --- | --- | --- | --- | --- | --- | --- |


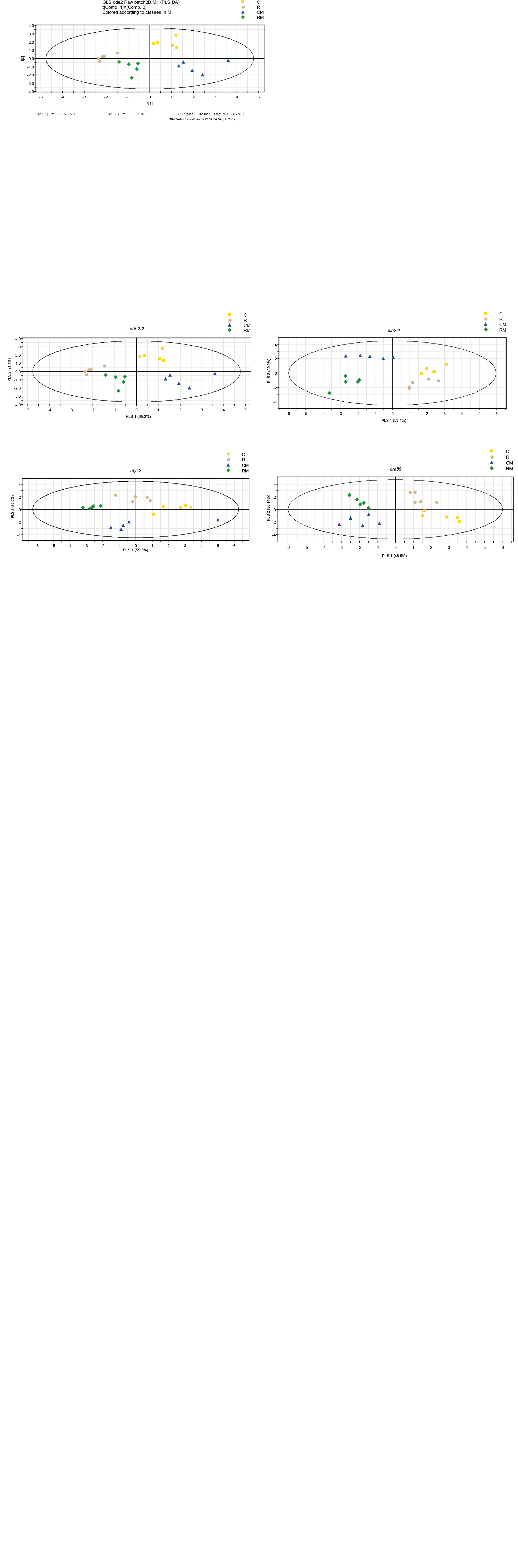


**Fig. S1** Projection to Latent Structures Discriminant Analysis (PLS-DA) comparison of *Arabidopsis thaliana* Col-0 GLS profile from the shoot of mutants *dde2-2*, *ein2-1*, *myc2*, *ora59*. Treatments are control plants **(C)**, rhizobacteria-treated plants **(R)**, control plants infested with *Mamestra brassicae* **(CM)**, or rhizobacteria-treated plants infested with *M. brassicae* **(RM).** Grouping pattern of samples according to the first two principal components and the Hotelling’s ellipse of the 95% confidence interval for the observations. Each point (*N* = 5 replicates) represents one sample from a pool of *A. thaliana* shoot collected from 5 plates.

**
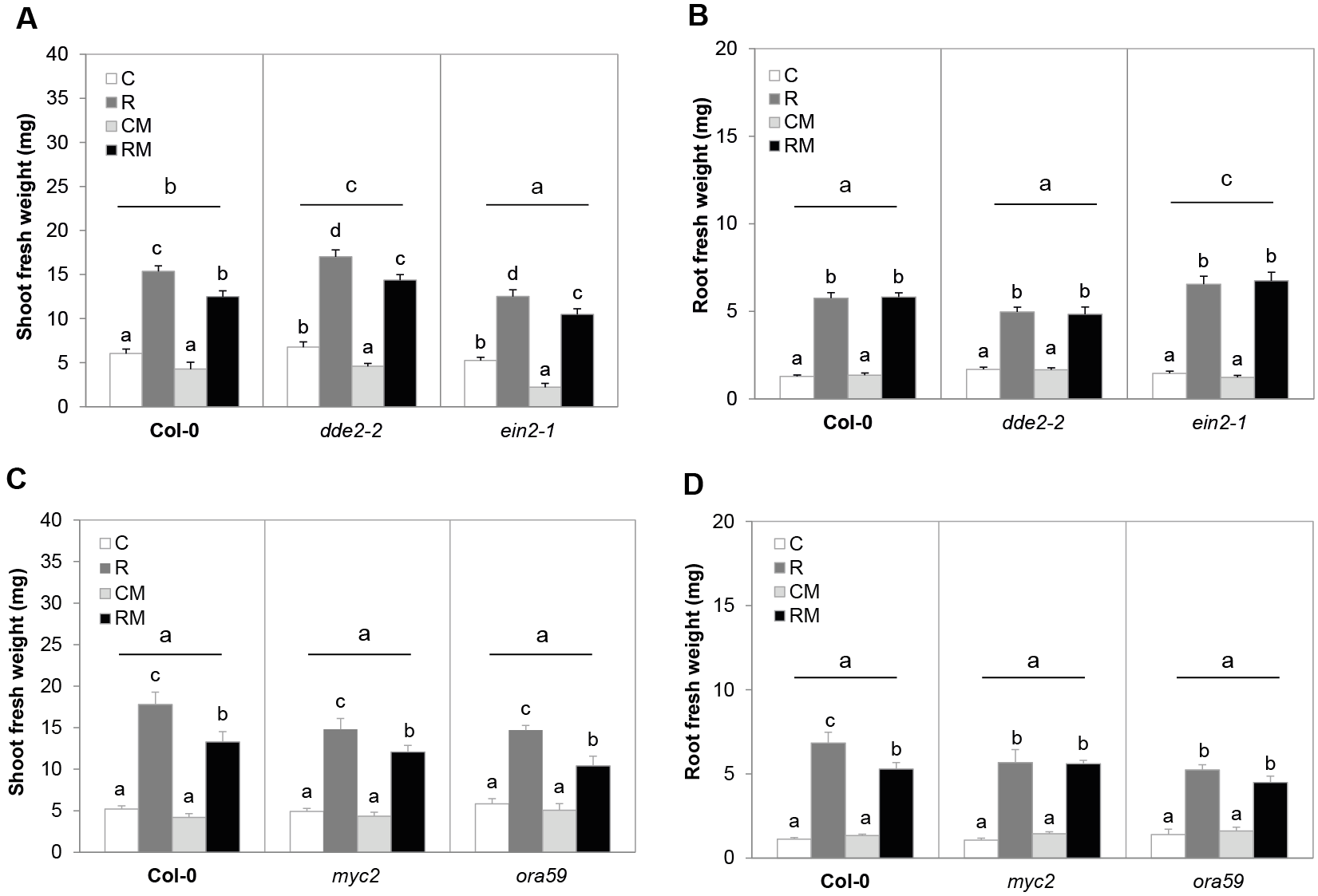
**

**Fig. S2** Shoot and root fresh weight (mean ± SE) of *Arabidopsis thaliana* Col-0, JA biosynthesis impaired mutant *dde2-2* and ethylene insensitive mutant *ein2-1*, **(A, B)** Col 0, *myc2*, *ora59* **(C, D)** of control plants (C), rhizobacteria-treated plants (R), control plants infested with *Mamestra brassicae* (CM), rhizobacteria-treated plants infested with *M. brassicae* (RM) (*N* = 6 to 10 replicates). Comparisons are within line (one-way ANOVA, LSD post hoc test, *P* < 0.05), and between lines (two-way ANOVA. LSD *post hoc* test, *P* < 0.05).
